# Subcutaneous Ligation

**Published:** 1892-08

**Authors:** B. P. McVey

**Affiliations:** Ella, Brazoria County, Texas


*Editor Daniel's Texas Medical Journal:*

### SUBCUTANEOUS LIGATION.

ELLA, BRAZORIA COUNTY, TEXAS, August 9, 1892.

*Editor Daniel's Texas Medical Journal:*

Prominent among the original communications in the last issue of the JOURNAL is an article by Dr. Thos. J. Pugh, entitled "Subcutaneous Ligature of an Artery." All I have to say on the subject will be through no sense of harsh criticism or disrespect to the originality of its author; on the contrary, I think every man should have enough individuality about him to think for himself, and then put his convictions into execution; and if his judgment has misguided he had better not say much about it, but if the experiment prove successful and your expectations are realized, do not hide your light under a bushel, but place it out on a high pinnacle (DANIEL'S TEXAS MEDICAL JOURNAL, or some like conspicuous place), that it may shed its rays of enlightenment throughout the land.

Prof. S. E. Chaille, M. D., of Tulane University, told his class once in my hearing, that the greatest drawback to the grogress of medical learning was the habit of following in the old trodden path. He says "If Austin Flint, Prof. Chaille or any body else tells you a thing is right, and it doesn't appeal to your reason as being that way, don't believe it until you have convinced yourself." Now, it is this principle I shall act upon while making a few remarks on Dr. Pugh's "Subcataneous Ligature of an Artery."

From a theoretical standpoint the operation is open to some objections.

In the first place, it looks to an impartial observer like a piece of bungling guess-work. Nerves, veins and arteries run the same course, and often in such close proximity as to render it impossible to go down blindfold and pick up the one without inclnding the other in the ligature.

Suppose we wanted to ligate the femoral artery one inch below Poupart's ligature where it is enclosed in a common fibrous sheath with the femoral vein, should we pass our curved needle armed with a deadly strand of silk around both vessels, insuring, you might say, gangrene and a consecutive hip-joint removal of the member?

Let us suppose another case, that to some of us might some day be more real than imaginary. Suppose we wanted to ligate the subclavian in its first part, where the phrenic nerve passes between the subclavian artery and subclavian vein, we would, I suppose, pass our curved needle clear around both vessels, including the phrenic nerve within its fatal grasp? The pneumogastric might also be included in a similar manner.

Another case and I will desist, though others might be mentioned.

Suppose we want to ligate an artery of one of the lower extremeties of an old person with feeble circulation, and happen to tie up a good sized vein with it, what could follow but gangrene? Again, Dr. Pugh says, tie up vessels, muscles, nerves and all together; that an occasional dose of morphine will allay the pain.

We all know the consequence of ligating a nerve stump after amputations, and I see no reason why it should not be as bad when tied in its continuity.

With all this staring a man in the face who theorizes before practicing, and thinks before acting, how can he speak these words: "In my opinion subcutaneous ligation should be resorted to in all cases where the artery can be reached without cutting'*?

To say the least, the procedure looks to be unnecessary, for if a vessel is near enough the surface to be reached with a needle, the traumatism produced by cutting down on it so that we can see what we are doing, is but little more than a skin wound and is hardly taken into account.

I see but one place wherein the operation might be admissible, and that would be in an old person with good circulation whose arteries are brittle from calcareous degeneration, then I believe it would be better to cut down and include such tissues as are necessary to pad the ligature, and not risk tying up veins and nerves.

B. P. McVey, M. D.

